# Risk Factors for Buruli Ulcer in Ghana—A Case Control Study in the Suhum-Kraboa-Coaltar and Akuapem South Districts of the Eastern Region

**DOI:** 10.1371/journal.pntd.0003279

**Published:** 2014-11-20

**Authors:** Ernest Kenu, Kofi Mensah Nyarko, Linda Seefeld, Vincent Ganu, Michael Käser, Margaret Lartey, Benedict Nii Laryea Calys-Tagoe, Kwodwo Koram, Richard Adanu, Oliver Razum, Edwin Afari, Fred N. Binka

**Affiliations:** 1 Korle-Bu Teaching Hospital, Department of Medicine–Fevers Unit, Korle-Bu, Ghana; 2 University of Ghana School of Public Health, Department of Epidemiology and Disease Control, Accra, Ghana; 3 Ghana Health Service, Disease Control and Prevention Department, Accra, Ghana; 4 University of Bielefeld, Department of Epidemiology, School of Public Health, Bielefeld, Germany; 5 Swiss Tropical and Public Health Institute, Basel, Switzerland; 6 University of Basel, Basel, Switzerland; 7 University of Ghana Medical School, Department of Medicine, Korle-Bu, Ghana; 8 Noguchi Memorial Institute of Medical Research, Department of Epidemiology, University of Ghana, Logan, Ghana; 9 University of Health and Allied Sciences, Department of Epidemiology and Disease Control, Ho, Ghana; Kwame Nkrumah University of Science and Technology (KNUST) School of Medical Sciences, Ghana

## Abstract

**Background:**

Buruli ulcer (BU) is a skin disease caused by *Mycobacterium ulcerans*. Its exact mode of transmission is not known. Previous studies have identified demographic, socio-economic, health and hygiene as well as environment related risk factors. We investigated whether the same factors pertain in Suhum-Kraboa-Coaltar (SKC) and Akuapem South (AS) Districts in Ghana which previously were not endemic for BU.

**Methods:**

We conducted a case control study. A case of BU was defined as any person aged 2 years or more who resided in study area (SKC or AS District) diagnosed according to the WHO clinical case definition for BU and matched with age- (+/−5 years), gender-, and community controls. A structured questionnaire on host, demographic, environmental, and behavioural factors was administered to participants.

**Results:**

A total of 113 cases and 113 community controls were interviewed. Multivariate conditional logistic regression analysis identified presence of wetland in the neighborhood (OR = 3.9, 95% CI = 1.9–8.2), insect bites in water/mud (OR = 5.7, 95% CI = 2.5–13.1), use of adhesive when injured (OR = 2.7, 95% CI = 1.1–6.8), and washing in the Densu river (OR = 2.3, 95% CI = 1.1–4.96) as risk factors associated with BU. Rubbing an injured area with alcohol (OR = 0.21, 95% CI = 0.008–0.57) and wearing long sleeves for farming (OR = 0.29, 95% CI = 0.14–0.62) showed protection against BU.

**Conclusion:**

This study identified the presence of wetland, insect bites in water, use of adhesive when injured, and washing in the river as risk factors for BU; and covering limbs during farming as well as use of alcohol after insect bites as protective factors against BU in Ghana. Until paths of transmission are unraveled, control strategies in BU endemic areas should focus on these known risk factors.

## Author Summary

*Mycobacterium ulcerans* is the causative agent of Buruli ulcer (BU) which affects the skin, can lead to extensive ulceration, and often results in disabilities. The exact mode of transmission of the disease is still unknown. Previous studies have identified demographic, socio-economic, health and hygiene, as well as environment, related risk factors for BU. This case-control study was done to ascertain the risk factors in a study area in Ghana which was previously non-endemic for BU. The study involved 226 participants, of which 50% were BU confirmed cases and 50% age-, gender-, and community matched controls (persons who had no signs or symptoms of active or inactive BU). This study found presence of wetland, insect bites in water, use of adhesive when injured, and washing in the Densu river as risk factors associated with BU. These factors were similar to previous studies and hence should be used in the implementation of national BU control strategies.

## Introduction

Buruli ulcer (BU) is a chronic debilitating skin disease caused by *Mycobacterium ulcerans* [1], [2]. BU depicts the third and second most common mycobacterial disease, globally and in Ghana, respectively [3], [4]. Currently, BU has been reported in over 30 countries in four continents [1], [2], [5], [6] but West Africa is the region most affected [1], [6]. The first case of BU in Ghana was reported in 1971 by Barley [7], [8], and ever since over 426 communities have reported cases. These communities are in the Ashanti, Brong Ahafo, Eastern, Greater Accra and Western regions. Amofah et al found the highest prevalence rate of 87.7/100,000 in the Ga West District [9].

Jacobsen and Padgett systematically reviewed extensive epidemiological studies done to identify risk factors associated with *M. ulcerans* throughout the world. The commonly reported risk factors associated with BU were slow flowing or stagnant water [4], [10]–[13], wading [14], [15] or washing clothes in swampy areas of slow flowing waters [16], and the use of short clothes during farming [15], [16]. Merritt et. al. reported similar risk factors in their systematic review on ecology and risk factors for transmission of BU [6]. Other risk factors reported were close proximity to human disturbed aquatic habitats [6], the use of unprotected water from swamps [17] and rivers [4], [7], and agricultural land use [18]. Reduced risk for BU, however was associated with the use of protected water sources in some settings [14], [17] as well as hygienic practices such as use of soap for bathing, use of alcohol to clean wounds, or injured sites and proper wound care [4], [14], [15]. Researchers in Amansie West District of Ghana demonstrated spatial relationship between BU prevalence and the immunosuppressant arsenic [13].

With regard to the role of insect bites in the transmission of *M. ulcerans*, water bug (aquatic *Hemipterans*) species have been particularly addressed [19], [20], [21], [22], [23]. Series of studies demonstrated mosquitoes and water bugs to carry *M. ulcerans* in endemic areas [24], [25]. Australian studies showed association of mosquito related risk factors with BU [26], [27], and experimental infection of mice bitten by infected water bugs in laboratory provided evidence to support their involvement [21], [28]. The argument for mosquitoes as vectors gained more ground when the use of bed nets was found to reduce the risk of BU [4], [15], [29]. Children aged less than fifteen years are overrepresented compared to adults albeit any age can be affected [6], [7], [30], [31]. Even though such risk factors have been identified, the exact mechanism by which humans contract BU in or near aquatic habitats is still not known. It has been hypothesized that *M*. *ulcerans* is transmitted through skin abrasions or skin injuries after contact with water, vegetation, or soil which still remains a hypothesis [18]. Without knowing the exact mode of transmission, the only recommendations to effectively prevent and control BU should be based on the currently known risk factors. SKC and AS Districts of the Eastern Region in Ghana have been recently identified as BU endemic but data on the prevailing risk factors was not yet available. Here, we conducted a case control study to identify the risk factors for BU in these previously non-endemic districts.

## Methods

### Study design and case definition

**Figure 1:**
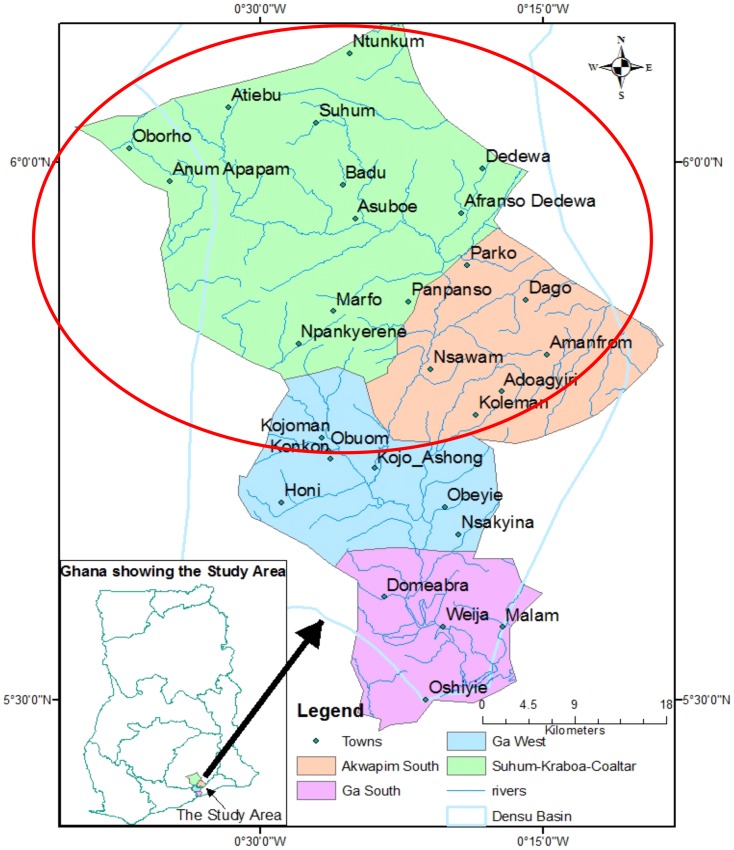
Map of the study area showing the Basin of the Densu River.

A case-control study was designed in two health districts, SKC and AS of the Eastern Region. The cases were identified through active community case search by trained Community Based Volunteers (CBVs) (Figure 1).

#### Cases

A probable case was defined as any person aged 2 years or more who resided in either the SKC or AS District presenting with active BU and clinically diagnosed between May 2010 to December 2011 by trained and specialized health professionals. A confirmed case was defined as a probable case with evidence of *M. ulcerans* infection by either positive polymerase chain reaction (PCR) or a positive Ziehl-Neelsen stain for acid-fast bacilli in smears of lesions. The laboratory confirmations were done at the Noguchi Memorial Institute for Medical Research (NMIMR), Accra, Ghana.

#### Controls

An eligible community control was defined as any person aged 2 years or more who resided in the community/neighbourhood where the case came from but did not have BU. Randomly selected controls were matched to cases for age (+/−5 years), gender, and community affiliation.

### Ethics statement

Information was provided to all members of the various communities and subsequently individually to the participants. Enrollment into the study was voluntary. All adult subjects provided written informed consent and a parent or guardian of any child participant provided written informed consent on their behalf. Ethical clearance was obtained from the NMIMR Institutional Review Board and the Ghana Health Service Ethical Committee. The approval was renewed yearly during the period of the study.

### Sample size

We used the power calculation tool (Epi Info software version 3.5.1) to determine the sample size. We set alpha to 0.05 and power equal to 80%. The districts reported 40% use of unprotected water. The minimum of the odds ratio (OR) for the association between cases and controls was set at 2.25. We obtained a sample size of 214 participants, made up of 107 cases and 107 controls.

### Data collection

BU active community case search was conducted by trained CBVs from May 2010 to December 2011. The research team was introduced to the head of the community, opinion leaders and solicited their cooperation on the research being carried out. Sensibilization was given to the community members through town cries “gongon beater” and by information using posters and pictures of BU prior to physical examination. Research assistants administered standardized questionnaires that covered issues on demography (age, gender, place of residence, marital status, occupation, and educational status), and behavioral activities (swimming, wading, fishing, wearing of protective clothing and personal hygiene). In addition, environmental issues (nearby presence of wetland/swamp, vegetations, cocoa or coffee plantations, sources of drinking water, type of houses, sharing of living space with animals/pets and other peculiar characteristics of the locations were also assessed. All questions were closed-ended and the questionnaires were verbally administered in English or the local language, Twi. Bacille Calmette-Guérin (BCG) vaccination was assessed by observing for the presence of the scar on the left shoulder around the deltoid region as vaccination cards were difficult to assess and in some cases missing. Wound swabs from ulcers and fine needle aspirates from nodules were used for laboratory confirmation.

### Data analysis

BU was the dependent variable and demographic, host related, environmental and behavioural factors as the independent variables. Significance level was set at a p-value less than 0.05. Univariate analysis was done using conditional logistic regression to calculate odds-ratios (OR) and 95% confidence intervals (95% CI) to explore the association between the exposure variables and BU. All variables obtained from the univariate analysis with p-values ≤0.1 were retained for the multivariate model. The variables in the final model were retained after a step-by-step backward elimination using multiple conditional logistic regression.

## Results

### Characteristics of cases

A total of 141 probable BU patients were enrolled, from which 113 (80.1%) were confirmed PCR positive. Among those, 66 (58.4%) were also positive for Ziehl-Neelsen stain. The median age of the confirmed cases was 28 years (ranging from 2 to 102 years). The commonest age group affected was above 24 years with 54.9% (62/113). Among the case patients 50.4% (57/113) were female and 49.6% (56/113) were male.

In addition to the various BU active lesions, contracture deformities were observed in twelve of the cases with active lesions, extensive scar due to BU in five of the cases and one patient had had amputation of the right little toe. Ethnic group distribution of the parents of the participants were Akan, Ewe and Ga Adangme. For parental ethnic groups 35.4% (40/113) of fathers of case patients were of Akan ethnic group, 50.4% (57/113) were Ewe and 14.2% (16/113) were Ga Adangme (Table 1).

**Table 1:** Characteristics of confirmed cases of BU in Suhum-Kraboa-Coaltar and Akuapem South Districts of the Eastern Region.

Characteristics	Confirmed BU Cases (n, %)	Characteristics	Confirmed BU Cases (n, %)
**N**	113 (100%)	**Lesion at time of presentation**	
**Sex**		** Papule**	0 (0.0)
** Female**	57 (50.40)	** Plaque**	0 (0.0)
** Male**	56 (49.6)	** Nodule**	18 (15.9)
		** Oedema**	0 (0.0)
**Age(median, range)**	28 (2–102)	** Ulcers**	95 (84.1)
** <10**	13 (11.5)		
** 10**–**14**	19 (16.8)	**Localization±**	
** 15**–**24**	19 (16.8)	** Lower limb**	76 (67.9)
** ≥24**	62 (54.9)	** Upper limb**	24 (21.4)
**Educational Status**		** Trunk (Breast)**	1 (0.9)
** No education**	31 (27.4)	** Head and Neck**	7 (6.2)
** Primary/Junior High School**	71 (62.8)	** Lower and upper limbs**	4 (3.6)
** Secondary/Tertiary**	11 (9.7)	**Classification of lesions**	
**Fathers Ethnic group**		** Category I**	18(15.9)
** Akan**	40 (35.4)	** Category II**	10 (8.9)
** Ewe**	57 (50.4)	** Category III**	85 (75.2)
** Ga Adangme**	16 (14.2)		

### Univariate analysis

#### Demographic factors

An association between the ethnic group of the parents of the case patients and the community controls was assessed and it was realized that, BU was less common in the Akan ethnic group compared to the Ewe and Ga Adangme. There was a significant association between level of education and risk of BU. Individuals with higher education were protected from developing BU as compared to those without education (Table 2).

**Table 2:** Univariate analysis of selected variables for BU in the Suhum-Kraboa-Coaltar and Akuapem South Districts of the Eastern Region, Ghana; Community-matched case control study.

Characteristic	No. (%) of Cases Subject (n = 113)	No. (%) of Control Subject (n = 113)	Univariate OR (95% CI)	P- Value
**Demographic**				
Ethnic group of the father Akan/Others	40 (35.4)	56 (49.6)	0.56(0.33–0.95)	0.04*
Ethnic group of the mother Akan/Others	41(36.3)	58 (51.3)	0.54(0.32–0.92)	0.03*
Education level: secondary or more/primary or nil	11 (9.7)	24 (21.2)	0.40(0.19–0.86)	0.03*
**Health**				
Bacillus Calmette Gu 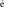 rin (BCG) Scar: Yes/No	86 (76.1)	95 (84.1)	0.60 (0.31–1.2)	0.18
History of TB: Yes/No	6 (5.3)	2 (1.8)	3.1 (0.62–15.8)	0.28
**Treatment when injured**				
Use of soap and water: Yes/No	21 (18.6)	50 (44.2)	0.29(0.16–0.53)	<0.001*
Rubbing the area with alcohol after insect bite: Yes/No	9 (8.0)	49 (43.4)	0.11 (0.05–0.25)	<0.001*
Use of leaves on injury site: Yes/No	74 (65.5)	45 (39.8)	2.9 (1.7–4.9)	<0.001*
Use of adhesive bandage: Yes/No	32 (28.3)	14 (12.4)	2.8 (1.4–5.6)	0.005*
**Water contact/activities**				
Bath for hygiene in open borehole: Yes/No	21 (18.6)	7 (6.2)	3.5(1.4–8.5)	0.008*
**Insect Bite/Behavior**				
Insect bite in water/mud Yes/No	96 (85.0)	7(6.19)	3.5 (1.8–6.6)	<0.001*
Use of bed net:Yes/No	87 (77.0)	77(68.1)	1.6 (0.87–2.8)	0.18
Use of mosquito coils: Yes/No	71 (62.8)	70(61.9)	1.0 (0.6–1.8)	1.0

*Significant association between variable and BU.

#### Health related factors

BCG scars were more frequently observed in the control group it was not significantly associated with BU (OR = 0.60, 95% CI = 0.31–1.2). Past history of tuberculosis and schistosomiasis were all not significantly associated with BU (Table 2).

Success with the type of treatment used when the subjects get injured considered the use of soap and water (OR = 0.29, 95% CI = 0.16–0.53), rubbing the area with alcohol when injured or after an insect bite (OR = 0.11, 95% CI = 0.05–0.25), use of leaves on injury site (OR = 2.9, 95% CI = 1.7–4.9) and use of adhesive bandage (OR = 2.8, 95% CI = 1.4–5.6) which were all associated with BU.

#### Insect bites/behavior

The case patients reported more frequently of an insect bites in water or wading in mud than the community controls did (OR = 3.5, 95% CI = 1.8–6.6). There was no significant association between the use of bed nets and BU (OR = 1.6, 95% CI = 0.87–2.8). The study found no significant association between the use of mosquito coils and BU (OR = 1.0, 95% CI = 0.6–1.8) (Table 2).

#### Environmental factors

Case patients reported less frequently of staying in mud wall house (OR = 0.41, 95% CI = 0.23–0.73) and around cocoa plantation in their immediate environment (OR = 0.55, 95% CI = 0.32–0.94) than the community controls did. However, the case patients were living near wetland (OR = 6.9, 95% CI = 3.8–12.4), had river(s) in their immediate neighborhood (OR = 2.5, 95% CI = 1.4–4.3), had bush in their immediate environment (OR = 2.7, 95% CI = 1.3–5.4), shared living space with poultry (OR = 1.9, 95% CI = 0.6–5.8), shared living space with cats (OR = 1.9, 95% CI = 1.1–3.3), and drank protected water (OR = 2.6, 95% CI = 1.2–5.7) (Table 3).

**Table 3:** Univariate analysis of selected variables for BU in the Suhum-Kraboa-Coaltar and Akuapem South Districts of the Eastern Region, Ghana; Community-matched case control study.

Characteristic	No. (%) of Case Subject (n = 113)	No. (%) of Control Subject (n = 113)	Univariate OR (95% CI)	P- Value
**Environment**				
Mud wall: Yes/No	68 (60.2)	89 (78.8)	0.41 (0.23–0.73)	0.004*
Presence of Cocoa Plantation in immediate neighborhood: Yes/No	36 (31.9)	52 (46.0)	0.55 (0.32–0.94)	0.04*
Presence of bush in immediate neighborhood: Yes/No	100 (88.5)	84 (74.3)	2.7 (1.3–5.4)	0.01*
Presence of wetland: Yes/No	87 (77.0)	37 (32.7)	6.9 (3.8–12.4)	<0.001*
Presence of river in the immediate neighborhood: Yes/No	81 (71.7)	57 (50.4)	2.5 (1.4–4.3)	0.002*
Share living space with poultry: Yes/No	85(75.2)	70 (61.9)	1.9(0.6–5.8)	0.04*
Share living space with cats:Yes/No	52 (46.0)	35 (31.0)	1.9 (1.1–3.3)	0.03*
Drinking water: protected/unprotected water	25(22.1)	11(9.7)	2.6(1.2–5.7)	0.02*
**Water contact/activities**				
Wading on the Densu river: Yes/No	68 (60.2)	34 (30.1)	3.5 (2.0–6.1)	<0.001*
Farming: Yes/NO	70 (61.9)	93 (82.3)	0.35 (0.19–0.65)	0.001*
No Farming	43 (38.1)	20 (17.7)	Reference	
Farming with long sleeves	47 (41.6)	89 (78.8)	0.25 (0.13–0.47)	<0.0001*
Farming with short sleeves	23 (20.4)	4 (3.5)	2.6 (0.76–11.9)	<0.07
No Farming	43 (38.1)	20 (17.7)	Reference	
Wearing of long pants to farm	60 (53.1)	91 (80.5)	0.31(0.16–0.57)	<0.0001*
Wearing of short pants to farm	10(8.9)	2 (1.8)	2.3 (0.43–23.5)	0.25

*Significant association between variable and BU.

### Water contact activities

Wading in the Densu river was more frequent among the case patients than the community controls and was significantly associated with BU (OR = 3.5, 95% CI = 2.0–6.1). However, wading in other rivers or streams, fetching of water and fishing in Densu river were not significantly associated with BU. Taking a bath with water taken from an open borehole was more frequent among case patients than community controls (OR = 3.5, 95% CI = 1.4–8.5).

### Farming activities

With no farming as a reference point, there was significant association between farming with long sleeves and BU (OR = 0.31, 95% CI = 0.16–0.57), long pants (OR = 0.25, 95% CI = 0.13–0.5) but not significantly associated with use of short sleeves and short pants when farming (Table 3).

### Multivariate analysis

Rubbing the area with alcohol after an insect bite (0.21; 95% CI = 0.008–0.57) and farming with long sleeve clothes (0.29; 95% CI 0.14–0.62) were found to be protective factors. Insect bite in water/mud (OR = 5.7, 95% CI = 2.5–13.1), presence of wetland (OR = 3.9, 95% CI = 1.9–8.2), use of adhesive bandage (OR = 2.7, 95% CI = 1.1–6.8), wading in Densu river (OR = 2.3, 95% CI = 1.1–4.96) and house wall built with mud (OR = 2.6, 95% CI = 1.1–5.9) were risk factors associated with BU (Table 4).

**Table 4:** Multivariate backward elimination model of conditional logistic regression for risk factors for BU in the Eastern Region, Ghana; Community-matched case control study.

CHARACTERISTIC	Multivariate OR (95% Cl)	P- VALUE
Presence of wetland	3.9 (1.9–8.2)	<0.001*
Insect bite in water/mud	5.7 (2.5–13.1)	<0.001*
Rubbing the area with alcohol after bite	0.21 (0.008–0.57)	0.002*
Use of adhesive bandage	2.7 (1.1–6.8)	0.035*
Washing in Densu river	2.3 (1.1–4.96)	0.028*
Farming clothes with long sleeves	0.29 (0.14–0.62)	0.001*
House wall built with mud	2.6 (1.1–5.9)	0.022*

*Statistically significant.

## Discussion

This study identified activities that showed statistically significant association with BU in SKC and AS Districts of the Eastern region of Ghana, an area recently identified as being endemic for BU. Farming with long sleeve clothes and rubbing an insect bite area with alcohol were associated with decreased risk of contracting BU. On the other hand, presence of wetland, insect bites in water/mud, washing in the Densu river, use of adhesive bandage and house walls built with mud were identified as risk factors for BU.

Without doubt, all limitations associated with the case control study approach apply to this investigation. Most of the case patients have been living with the disease for more than two years, hence prevalent cases rather than incident cases were recruited. For a chronic and rare disease like BU, association of disease persistence may be confounded with disease development. Also, recall bias remained a major limitation to this study, both from case patients and respondent parents on behalf of their wards. However, the interviewers were trained to ensure that appropriate responses were elicited from the respondents so as to minimize any form of bias or confounding effects to the findings.

This study comes sequent to several epidemiological studies identifying risk factors for transmission of BU [4], [7], [13], [14], [15], [25], [29], [32], [33], [34], and our findings validate in the Eastern Region of Ghana what has been reported in other countries. Ulcerative forms of disease presentation constituted 84% (95/113) of all cases. This implies that most of the case patients presented or were diagnosed late, probably due to factors such as transportation costs, feeding costs, and productivity loss [3], [35], [36]. This may be the underlying reason for the high median age of the participants in the study. It was found that 67.9% (76/113) of the case patients had lesions on their lower limbs [15], [37]–[39] albeit with no preference to either side of the body. This is in contrast to an earlier study done in the Ashanti Region of Ghana reporting more frequent affection of the left leg [24], a finding which could also not be confirmed by other studies [37], [40].

Concerning earlier findings of predisposition for or genetic link to BU [33] the present result show (albeit not significant in the multivariate model) that BU was less common in the Akan ethnic group.

No significant relationship was found between anamnesis of a past tuberculosis [15] nor to a protective role of BCG vaccination to BU, as indicated by previous reports [4], [15], [26], [30], [41], [42].

Case patients reported more frequently insect bites in water or wading in mud than the community controls did, which was evident as statistically significant in other studies [15], [26]. This finding tends to support the hypothesis that *M. ulcerans* can only enter the body through broken skin due to either insects bites or abrasions. Likewise, an appropriate initial treatment upon injury like rubbing the area with alcohol seems to offer protection against development of BU. Surprisingly, the use of adhesive bandage when hurt increased the odds of contracting BU, probably owing to the fact that often adhesive bandages were already being used by other persons and thus contaminated. In fact, most such bandages looked old and dirty. Wading, swimming, and fishing in the Densu river were not identified as risk factors for BU. Swimming was not widely practiced in the study area [7] although a study in Cote d'Ivoire found such an association [16]. The type of fishing undertaken in the Eastern Region of Ghana differs from habits in many areas that did identify correlations to fishing activities [4], [15], [16]. Here, commonly either lines with hooks or small nets are being placed at the bank of the river hence resulting in little or no contact to water.

The present study confirms, however, findings of other studies [4], [15], [16] that arming with long sleeves and long pants protects against BU. Long clothes may protect from small injuries or insect bites as possible means of entry for *M. ulcerans*.

In line with previous studies, the use of soap for washing was found to be associated with a decreased risk of *M. ulcerans* infection [4], [14]. In order to approach the role of mosquitoes in the transmission of BU, we used the protection of bed nets as a proxy to assess association to contracting BU. In accordance with Raghunathan's finding [4], this study showed no evidence for protective effects of bed net usage. Since other studies showed the contrary [14], [15], [26] we reason that in malaria endemic countries, the role of mosquitoes in the transmission of BU may be under investigated. Likewise, and also in contrast to earlier reports [4], [15], the present study showed no evidence of association between the use of mosquito coils and BU.

In this newly identified BU endemic area of the SKC and AS Districts in the Eastern Region of Ghana, our study identified as risk factors the presence of wetlands, insect bites in water, use of adhesive when injured and washing in the Densu river. In contrast, covering limbs during farming and use of alcohol after insect bites were found to be protective factors for BU. Until the mode of transmission is completely unraveled, provision of information in public health measures and steadily raising awareness of these risk factors are important means to both prevent and control BU.

## Supporting Information

Table S1 Non BU characteristics in the BU study in Suhum-Kraboa-Coaltar and Akuapem South Districts of the Eastern Region.
(DOCX)

Table S2 Univariate analysis of selected variables for BU in Suhum-Kraboa-Coaltar and Akuapem South Districts of the Eastern Region, Ghana; Community-matched case-control study.
(DOCX)

Checklist S1 STROBE statement—Checklist on *case-control studies*.
(PDF)

Questionnaire S1 Questionnaire for risk factors for transmission of *M. ulcerans* in Suhum-Kraboa-Coaltar and Akuapem South Districts of the Eastern Region, Ghana.
(PDF)
