# Supplementary material for: Risk Factors for Buruli Ulcer in Ghana—A Case Control Study in the Suhum-Kraboa-Coaltar and Akuapem South Districts of the Eastern Region
Source: PLoS Negl Trop Dis. 2014 Nov 20;8(11):e3279. doi: 10.1371/journal.pntd.0003279 (PMC4238991; doi:10.1371/journal.pntd.0003279)
Supplement: Table S1 — Non BU characteristics in the BU study in Suhum-Kraboa-Coaltar and Akuapem South Districts of the Eastern Region. (DOCX) [file pntd.0003279.s001.docx]

**Non BU Characteristics in Buruli Ulcer Study in Akuapem South and Suhum-Kraboa-Coaltar Districts of Eastern Region**

| **Characteristics** | **Not Buruli Cases (n, %)** | **Characteristics** | **Not Buruli Cases (n, %)** |  |
| --- | --- | --- | --- | --- |
| **N** | 28 (19.9) | **First Lesion** |  |  |
| **Sex** |  | **Papule** | 0 (0) |  |
| **Female** | 11 (39.3) | **Plaque** | 0 (0) |  |
| **Male** | 17 (60.7) | **Nodule** | 27 (96.4) |  |
|  |  | **Oedema** | 1 (3.6) |  |
| **Age( median, range)** | 29 (2 -98) | **Active Ulcers** | 0 (0) |  |
| **<10** | 6 (21.4) | **Don’t Know** | 0 (0) |  |
| **10-14** | 8 (28.6) | **Localization±** |  |  |
| **15-24** | 6 (21.4) | **Leg** | 18 (64.3) |  |
| **≥ 24** | 8 (28.6) | **Arm** | 8 (28.5) |  |
| **Educational Status** |  | **Trunk (Breast)** | 0 (0) |  |
| **No education** | 8 (28.6) | **Head and Neck** | 1(3.6) |  |
| **Primary/Junior High School** | 17 (60.7) | **Leg and Arm** | 1 (3.6) |  |
| **Secondary/Tertiary** | 3 (10.7) | **Distal (D) part of the body** | 18 (64.3) | |
| **Fathers Ethnic group** |  | **Proximal (P) part of the body** | 9 (32.1) | |
| **Akan** | 12 (42.9) | **Both (D&P)** | 1 (3.6) | |
| **Ewe** | 8 (28.6) | **Right side of the body** | 14 (50) | |
| **Ga Adangme** | 8 (28.6) | **Left side of the body** | 12 (42.9) | |
|  |  | **Both (Right and left )** | 2 (7.1) | |
|  |  |  |  |  |
