# Supplementary material for: Risk Factors for Buruli Ulcer in Ghana—A Case Control Study in the Suhum-Kraboa-Coaltar and Akuapem South Districts of the Eastern Region
Source: PLoS Negl Trop Dis. 2014 Nov 20;8(11):e3279. doi: 10.1371/journal.pntd.0003279 (PMC4238991; doi:10.1371/journal.pntd.0003279)
Supplement: Table S2 — Univariate analysis of selected variables for BU in Suhum-Kraboa-Coaltar and Akuapem South Districts of the Eastern Region, Ghana; Community-matched case-control study. (DOCX) [file pntd.0003279.s002.docx]

**Table S1: Univariate analysis of selected variables for Buruli ulcer in Akuapem South and Suhum-Kraboa-Coaltar Districts of Eastern Region,Ghana; Community-matched case-control study**

| **Characteristic** | **No. (%) of Cases Subject (n=113)** | **No. (%) of Control Subject (n=113)** | **Univariate OR (95% CI)** | **P- Value** |
| --- | --- | --- | --- | --- |
| **Demographic** |  |  |  |  |
| No. of people in HH:<8/≥8 | 61 (54.0) | 45 (39.8) | 0.75 (0.41-1.4) | 0.44 |
| Expenditure: ≥ than $5/≤ $5 | 40 (35.4) | 49 (43.4) | 0.72 (0.42-1.2) | 0.28 |
| **Health** |  |  |  |  |
| Family history of TB: Yes/No | 9 (8.0) | 4 (3.5) | 2.4(0.71-7.9) | 0.25 |
| History of blood in urine: Yes/No | 24 (21.2) | 18 (15.9) | 1.4 (0.72-2.8) | 0.39 |
| **Environment** |  |  |  |  |
| Mud floor: Yes/No | 42 (37.2) | 52 (46.0) | 0.69(0.41-1.18) | 0.22 |
| Presence of Coffee Plantation in immediate neighborhood: Yes/No | 1 (0.9) | 3 (2.7) | 3.1 (0.31-29.8) | 0.62† |
| Presence of wood in immediate neighborhood: Yes/No | 97 (85.8) | 92 (81.4) | 1.4 (0.68-2.8) | 0.47 |
| Share living space with goat: Yes/No | 67 (59.3) | 59 (52.2) | 1.3 (0.79-2.26) | 0.35 |
| Share living space with pigs: Yes/No | 9 (8.0) | 5 (4.4) | 1.9 (0.6-5.8) | 0.41 |
| Share living space with dogs : Yes/No | 42 (37.2) | 49 (43.4) | 0.77 (0.45-1.3) | 0.42 |
| **Insect Bite/Behavior** |  |  |  |  |
| Use of bed net :Yes/No | 87 (77.0) | 77(68.1) | 1.6 (0.87-2.8) | 0.18 |
| Use of mosquito coils: Yes/No | 71 (62.8) | 70(61.9) | 1.0 (0.6-1.8) | 1.0 |
|  |  |  |  |  |

*Significant association between variable and buruli ulcer

| **Table S2: Univariate analysis of selected variables for Buruli ulcer in Akuapem South and Suhum-Kraboa-Coaltar Districts of Eastern Region, Ghana; Community-matched case-control study** | | | | |
| --- | --- | --- | --- | --- |
| **Characteristic** | **No. (%) of Case Subject (n=113)** | **No. (%) of Control Subject (n=113)** | **Univariate OR (95% CI)** | **P- Value** |
| **Water contact/activities** |  |  |  |  |
| Wading in river or stream: Yes/No | 46 ( 40.7) | 36 (31.9) | 1.5 (0.85-2.5) | 0.2 |
| Washing of clothes: Yes/No | 103 (91.2) | 107 (94.7) | 0.5 (0.2-1.6) | 0.44 |
| Fetching of water: Yes/No | 74 (65.5) | 70 (61.9) | 1.2 (0.68-2.0) | 0.68 |
| Do you fish: Yes/No | 16 (14.2) | 13 (11.5) | 1.3 (0.58-2.8) | 0.7 |
| Fishing in Densu river: Yes/No | 11 (9.7) | 8 (7.1) | 1.4 (0.55-3.7) | 0.63 |
| Do not Fish | 97 (85.8) | 100 (88.5) | Reference |  |
| Fish with long sleeves | 6 (5.3) | 5 (4.4) | 1.2 (0.35-4.5) | 0.37 |
| Fish with short sleeves | 10 (8.9) | 8 (7.1) | 1.3 (0.48-3.5) | 0.31 |
| Do not fish | 97 (85.8) | 100 (88.5) | Reference |  |
| Fish with long pants | 8 (7.1) | 8 (7.1) | 1.0 (0.36-2.8) | 0.48 |
| Fish with short pants | 5 (0.04) | 8 (7.1) | 1.7 (0.51-5.7) | 0.21 |
| Bath for hygiene: Yes/No | 112 (99.1) | 113 (100) |  | 1.0 |
| Bath for hygiene , but not in Densu: Yes/No | 97 (85.8) | 103 (91.2) | 0.59 (0.26-1.4) | 0.3 |
| Bath in Densu for hygiene : Yes/No | 25 (22.1) | 19 (16.8) | 1.4 (0.7-2.7) | 0.4 |
| Bath for hygiene, not in open borehole: Yes/No | 102 (90.3) | 103 (91.2) | 0.9 (0.37-2.2) | 1.0 |
| Swim/dive/playing in water: Yes/No | 32( 28.3) | 20(17.7) | 1.8 (0.98-3.5) | 0.08 |
| Swimming: Yes/No | 34 (30.1) | 28 (24.8) | 1.3 (0.73-2.3) | 0.46 |
| Swimming but not in Densu: Yes/No | 20 (17.7) | 14 (12.4) | 1.5 (0.73-3.2) | 0.35 |
| Swimming in Densu: Yes/No | 24 (21.2) | 16 (14.2) | 1.6 (0.82-3.3) | 0.22 |
