# Supplementary material for: Risk Factors for Buruli Ulcer in Ghana—A Case Control Study in the Suhum-Kraboa-Coaltar and Akuapem South Districts of the Eastern Region
Source: PLoS Negl Trop Dis. 2014 Nov 20;8(11):e3279. doi: 10.1371/journal.pntd.0003279 (PMC4238991; doi:10.1371/journal.pntd.0003279)
Supplement: Checklist S1 — STROBE statement—Checklist on case-control studies. (PDF) [file pntd.0003279.s003.pdf]

Checklist S1: STROBE Statement—Checklist of items that should be included in reports of ***case-control studies***

	Item No	Recommendation
**Title and abstract**	1	(*a*) Indicate the study's design with a commonly used term in the title or the abstract [**Within method section of the abstract page 2**]
		(*b*) Provide in the abstract an informative and balanced summary of what was done and what was found **[See results section of abstract page 2]**
**Introduction**		
Background/rationale	2	Explain the scientific background and rationale for the investigation being reported **[Within introduction section Page 4 and 5]**
Objectives	3	State specific objectives, including any prespecified hypotheses **[Within introduction section Page 5]**
**Methods**		
Study design	4	Present key elements of study design early in the paper **[Within the Methods section page 6]**
Setting	5	Describe the setting, locations, and relevant dates, including periods of recruitment, exposure, follow-up, and data collection **[Within the Methods section page 6]**
Participants	6	(*a*) Give the eligibility criteria, and the sources and methods of case ascertainment and control selection. Give the rationale for the choice of cases and controls **[Within the Methods section page 6]**
		(*b*) For matched studies, give matching criteria and the number of controls per case **[Within the Methods section page 6]**
Variables	7	Clearly define all outcomes, exposures, predictors, potential confounders, and effect modifiers. Give diagnostic criteria, if applicable **[Within the Methods section page 7]**
Data sources/ measurement	8*	For each variable of interest, give sources of data and details of methods of assessment (measurement). Describe comparability of assessment methods if there is more than one group **[Within the Methods section pages 6,7,8]**
Bias	9	Describe any efforts to address potential sources of bias**[Within the Methods section pages 6,7 and 8]**
Study size	10	Explain how the study size was arrived at [**Within the Methods section page 7]**
Quantitative variables	11	Explain how quantitative variables were handled in the analyses. If applicable, describe which groupings were chosen and why [**Within the Methods section pages 7 and 8**]
Statistical methods	12	(*a*) Describe all statistical methods, including those used to control for confounding [**Within the Methods section pages 7 and 8]**
		(*b*) Describe any methods used to examine subgroups and interactions **[Within the Methods section pages 7 and 8]**
		(*c*) Explain how missing data were addressed **[N/A]**
		(*d*) If applicable, explain how matching of cases and controls was addressed **[Within the Methods section page 6,7 and 8]**
		(*e*) Describe any sensitivity analyses **[N/A]**
**Results**		
Participants	13*	(a) Report numbers of individuals at each stage of study—eg numbers potentially eligible, examined for eligibility, confirmed eligible, included in the study, completing follow-up, and analysed **[Within the Results section pages 9and 10]**
		(b) Give reasons for non-participation at each stage **[N/A]**
		(c) Consider use of a flow diagram **[N/A]**
Descriptive data	14*	(a) Give characteristics of study participants (eg demographic, clinical, social) and information on exposures and potential confounders **[Within the Results section pages 9and 10]**
		(b) Indicate number of participants with missing data for each variable of interest **[N/A]**

Outcome data	15*	Report numbers in each exposure category, or summary measures of exposure **[N/A]**
Main results	16	(*a*) Give unadjusted estimates and, if applicable, confounder-adjusted estimates and their precision (eg, 95% confidence interval). Make clear which confounders were adjusted for and why they were included **[Within the Results section pages 12, 13and Tables 3-7]**
		(*b*) Report category boundaries when continuous variables were categorized **[Within the Results section pages 9, 10 and Table 1]**
		(*c*) If relevant, consider translating estimates of relative risk into absolute risk for a meaningful time period **[N/A]**

Other analyses	17	Report other analyses done—eg analyses of subgroups and interactions, and sensitivity analyses **[N/A]**
**Discussion**		
Key results	18	Summarise key results with reference to study objectives **[Within the Discussion section pages 13 and Table 7]**
Limitations	19	Discuss limitations of the study, taking into account sources of potential bias or imprecision. Discuss both direction and magnitude of any potential bias [**Within the Discussion section pages 13 and 14]**
Interpretation	20	Give a cautious overall interpretation of results considering objectives, limitations, multiplicity of analyses, results from similar studies, and other relevant evidence **[Within the Discussion section pages 13-17]**
Generalisability	21	Discuss the generalisability (external validity) of the study results **[Within the Discussion section pages 17]**
**Other information**		
Funding	22	Give the source of funding and the role of the funders for the present study and, if applicable, for the original study on which the present article is based **[Page 18]**

*Give information separately for cases and controls.

Note: An Explanation and Elaboration article discusses each checklist item and gives methodological background and published examples of transparent reporting. The STROBE checklist is best used in conjunction with this article (freely available on the Web sites of PLoS Medicine at http://www.plosmedicine.org/, Annals of Internal Medicine at http://www.annals.org/, and Epidemiology at http://www.epidem.com/). Information on the STROBE Initiative is available at http://www.strobe-statement.org.
