# Supplementary material for: Risk Factors for Buruli Ulcer in Ghana—A Case Control Study in the Suhum-Kraboa-Coaltar and Akuapem South Districts of the Eastern Region
Source: PLoS Negl Trop Dis. 2014 Nov 20;8(11):e3279. doi: 10.1371/journal.pntd.0003279 (PMC4238991; doi:10.1371/journal.pntd.0003279)
Supplement: Questionnaire S1 — Questionnaire for risk factors for transmission of M. ulcerans in Suhum-Kraboa-Coaltar and Akuapem South Districts of the Eastern Region, Ghana. (PDF) [file pntd.0003279.s004.pdf]

Questionnaire S1 :QUESTIONNIARS FOR RISK FACTORS FOR TRANSMISSION OF MYCOBACTERIUM ULCERANS IN SUHUM- KRABOA-COALTAR AND AKUAPEM SOUTH DISTRICTS OF EASTERN REGION, GHANA.

Form number ☐☐☐ **please circle the Appropriate Response** **MFORMNUM**

**Q.N⁰**	**Questions and filters**	**Answers**	**Codes**	**Skips**	**FIELDS**
1.	ID	☐☐☐☐☐			ID
2.	Date of interview	☐☐☐☐☐☐☐☐			DINT
3.	Do you have BU?<br>***(Wo wɔ Buruli Ulcer?)***	Yes (Aane)<br>No(Daabi)	1<br>2	 >>7	BUD
4.	Has the BU been confirmed?<br>***(Wɔn asi no pi sɛ wo wɔ Buruli ulcer)***	Yes (Aane)<br>No (Daabi)	1<br>2		CON
5a.	What form was the first lesion?<br>***(Yareɛ yi rehyɛ aseɛ no, na ɛte sɛn?)***	Papule<br>Nodule<br>Plaque<br>Edema<br>Active Ulcers	1<br>2<br>3<br>4<br>5		LES
5b.	What form is the lesion now?<br>***(Sesei yareɛ no te sɛn?)***	Papule<br>Nodule<br>Plaque<br>Edema<br>Active Ulcers<br>Old case (healed lesion)	1<br>2<br>3<br>4<br>5<br>6	} >> 6 (codes 1–5)<br>>>5c	

5c.	How old is the healed lesion(Old case)	_____________			
6.	Which part of the body is the lesion located?<br>***(Wo honam henfa na saa akuro yi da?)***	Leg<br>Arm<br>Trunk<br>Head<br>Distal<br>Proximal/Trunk/Head)<br>Right side<br>Left side	1<br>2<br>3<br>4<br>5<br>6<br>7<br>8		LOC
7.	How old are you? (Age in years)<br>***(Wɔn di mfeɛ sɛn?)***	☐☐			AGEC
8.	Gender ***(W'abɔsuo ne sɛn?)***	Male<br>Female	1<br>2		SEX
9.	What is the ethnic group of your father?<br>***(Wo papa yɛ deɛn nii?)***	Ga/Adangme<br>Ewe<br>Akan<br>Others	1<br>2<br>3<br>4		ETHF
10.	What is the ethnic group of your mother?<br>***(Wo maam yɛ deɛn ni?)***	Ga/Adangme<br>Ewe<br>Akan<br>Others	1<br>2<br>3<br>4		ETHM
11.	What is your educational level?<br>***(W'akɔ sukuu aduru sɛn?)***	No Education<br>Primary/JHS<br>Secondary	1<br>2<br>3		EDUC

		Tertiary	4		
12.	What is your marital status?<br>***(W'awareɛ gyina pɛn yɛ dɛn?)***	Married<br>Not married	1<br>2		MAR
13.	How much money do you spend in this household per month? ***(Bosome biara, sika sɛn na mo de hwɛ fie?)***	Gh¢………………..per month			HHS
	**HEALTH**				
14.	Do you have a BCG scar? Look on the left shoulder.<br>***(Wo wɔ BCG nsaman wa kɔtwa no bi wɔ wo ho? Hwɛ w'abɛti a ɛwɔ wo nifa so).***	Yes (Aane)<br>No (Daabi)	1<br>2		BCG
15.	Have you had tuberculosis before? ***(W'anya nsamanwa da?)***	Yes (Aane)<br>No(Daabi)	1<br>2		HTB
16.	Do you have a family history of tuberculosis?<br>***(W'abusua mu no, yareɛ nsamanwa yi yɛ adeɛ a obi anya yareɛ yi bi da?)***	Yes (Aane)<br>No (Daabi)	1<br>2		FHTB
17.	Have you ever had blood in urine before?<br>***(W'adwonsɔ a mogya wɔ mu da?)***	Yes (Aane)<br>No(Daabi)	1<br>2		BLD
	**HOUSEHOLD / ENVIRONMENT**				
18.	Is the wall of your house made of Mud?<br>***(Wo fie afasuo no nɛteɛ na wɔde ayɛ anaa?)***	Yes (Aane)<br>No(Daabi)	1<br>2		WAL
19.	Is the floor of your house made of Mud?<br>***(Fie no fɔm no, wɔde nɛteɛ na ayɛ anaa?)***	Yes (Aane)<br>No(Daabi)	1<br>2		FLO

20.	How many people are in your household?<br>***(Nipa sɛn na ɛte wo fie?)***	_______________			HHN
21.	Do you have Cocoa plantation in your immediate neighborhood?<br>***(Kookoofuo bɛn wo mpɔtam ha anaa?)***	Yes (Aane)<br>No(Daabi)	1<br>2		COA
22.	Do you have Coffee plantation in your immediate neighborhood?<br>***(Coffeefuo bi bɛn wo mpɔtam ha anaa?)***	Yes (Aane)<br>No(Daabi)	1<br>2		COF
23.	Do you have bush in your immediate neighborhood?<br>***(Burɔnoo a wo te no, nwura afu wɔ anaa?)***	Yes (Aane)<br>No(Daabi)	1<br>2		BUS
24.	Do you have woods in your immediate neighborhood?<br>***(Wɔ burɔnoo no so yɛ nnuam anaa?)***	Yes (Aane)<br>No(Daabi)	1<br>2		WOD
25.	Do you have a wetland (Swamp) that is an area of land, usually fairly large, that is always wet and is overgrown with various shrubs and trees in your immediate neighborhood?<br>(***Wo burunoo yi so ha, aforɔ wɔ ha a kɛsɛ a nwura ahodoɔ fu wɔ mu anaa?)***	Yes (Aane)<br>No(Daabi)	1<br>2		SWP
26.	Do you have river in your immediate neighborhood?<br>***(Mo wɔ nsubɔntene wɔ mo mpɔtam ha?)***	Yes (Aane)<br>No(Daabi)	1<br>2		RIV
27.	Do you Share living space (that is staying under the same roof) with goats?<br>***(Wo yɛn mpɔnkye a wo ne wɔn na ɛte anaa?)***	Yes (Aane)<br>No(Daabi)	1<br>2		GOT

28.	Do you Share living space, (that is staying under the same roof) with poultry?<br>***(Wo yɛn nkokɔ a wo ne wɔn na ɛte anaa?)***	Yes (Aane)<br>No(Daabi)	1<br>2		POU
29.	Do you Share living space , (that is staying under the same roof) with pigs?<br>***(Wo yɛn mprɛko a wo ne wɔn na ɛte anaa?)***	Yes (Aane)<br>No(Daabi)	1<br>2		PIG
30.	Do you Share living space , (that is staying under the same roof) with cats?<br>***(Wo ne nkra na ɛte anaa?)***	Yes (Aane)<br>No(Daabi)	1<br>2		CAT
31.	Do you Share living space , (that is staying under the same roof) with dogs?<br>***(Wone nkraman na ɛte anaa?)***	Yes (Aane)<br>No(Daabi)	1<br>2		DOG
32.	What is your primary source of drinking water?<br>***(Wo nsuo a wonum no, henfa na wo nya firi?)***	River or stream<br>Borehole<br>Pipe borne	1<br>2<br>3		DRK
	**INSECT BITES/BEHAVIOR**				
33.	Have you received insect bite in water/mud ?<br>***(Ntumoa, wɔfiri nsuo anaa atɛkyɛ mu aka wo da?)***	Yes (Aane)<br>No(Daabi)	1<br>2		BIT

34.	Do you use bed nets?<br>***(Wo da ntontom dan mu?)***	Yes (Aane)<br>No(Daabi)	1<br>2		NET
35.	Do you Use mosquito coils?<br>***(Wo taa hye ntontom coil?)***	Yes (Aane)<br>No(Daabi)	1<br>2		COIL
	**TREATMENT WHEN HURT**				
36.	Do you use Soap and water?<br>***(Sɛ wo pira a, wo de nsuo ne samina hohoro?)***	Yes (Aane)<br>No(Daabi)	1<br>2		SOAP
37.	Do you Rub the area with alcohol after a bite?<br>***(Sɛ aboa bi ka wo a, wo de nsa twetwere so?)***	Yes (Aane)<br>No(Daabi)	1<br>2		ACH
38a.	Do you use Leaves on the site of injury?<br>***(Ɛyɛ a, wo pira a, wode ahahanman twetwere so?)***	Yes (Aane)<br>No(Daabi)	1<br>2		LEAV
38b.	What is the name of the leaves you use?<br>***(Nhahanma no, wɔfrɛ no sɛn)***	________________________			
39.	Do you use adhesive bandage when you get hurt?<br>***(Wo pira a, wo de bandage kyekyere so?)***	Yes (Aane)<br>No(Daabi)	1<br>2		BAN
	**ACTIVITIES**				
40.	Do you Wade in the Densu river?<br>***(Ɛyɛ a, wo kɔ nante, nante Densu nsuo no mu?)***	Yes (Aane)<br>No(Daabi)	1<br>2		DEN

41.	Do you Wade in a river or stream?<br>(***Ɛyɛ a, wo nante,nante anaa nsubɔntene mu?)***	Yes (Aane)<br>No(Daabi)	1<br>2		WRIV
42.	Do you Wash your clothes?<br>***(Ɛyɛ a wosi wo nneɛma?)***	Yes (Aane)<br>No(Daabi)	1<br>2		WAS
43.	Do you Fetch water?<br>(***Ɛyɛ a wokɔ nsuo?)***	Yes (Aane)<br>No(Daabi)	1<br>2		FET
44.	Do you Farm?<br>***(Wo yɛ afuo?)***	Yes (Aane)<br>No(Daabi)	1<br>2	If no >>49	FARM
45.	Do you Farm and wear long upper body clothing/shirt?<br>***(W'afuom ntaadeɛ a, ɛyɛ a wohyɛ wɔ wo soro no, ɛyɛ deɛ ne nsa woware?)***	Yes (Aane)<br>No(Daabi)	1<br>2		WEAR
46.	Do you Farm and wear short upper body clothing/shirt?<br>***(W'afuom ntaadeɛ a, ɛyɛ a wohyɛ wɔ wo yɛ deɛ ne nsa ɛyɛ ntiantɛa?)***	Yes (Aane)<br>No(Daabi)	1<br>2		WSHT
47.	Do you Farm and wear long pants/dress?<br>***(Worekɔ afuom a, wohyɛ ataadeɛ a, ware?)***	Yes (Aane)<br>No(Daabi)	1<br>2		WLOG
48.	Do you Farm and wear short pants/dress?<br>***(Worekɔ afuom a, wohyɛ ataadeɛ a ɛyɛ tia?)***	Yes (Aane)<br>No(Daabi)	1<br>2		FWSHT
49.	Do you Fish?<br>(***Wo yi nam?)***	Yes (Aane)<br>No(Daabi)	1<br>2	If no >>56	FISH

50.	Do you Fish, but not in the Densu river? ***(Wo yi nam nam, nanso Densu nsuo mu?)***	Yes (Aane)<br>No(Daabi)	1<br>2		NDEN
51.	Do you Fish in the Densu river? (***Wo yi nam wɔ Densu nsuo mu?)***	Yes (Aane)<br>No(Daabi)	1<br>2		INDEN
52.	Do you Fish with long upper body clothing? ***(Wo ntaadeɛ a wo hyɛ wɔ soro yi nam no, ɛyɛ a, na ɛware?)***	Yes (Aane)<br>No(Daabi)	1<br>2		FLCTH
53.	Do you Fish with short/no upper body clothing? ***(Wo ntaadeɛ a wo hyɛ wɔ soro de yi nam no yɛ a na ɛyɛ tia anaa wonhyɛ bi koraa?)***	Yes (Aane)<br>No(Daabi)	1<br>2		FSHTC
54.	Do you Fish with long lower body clothing? ***(Wo ntaadeɛ a, wo hyɛ wɔ fɔm de yin am no, yɛ a na ɛware anaa wonhyɛ bi koraa?)***	Yes (Aane)<br>No(Daabi)	1<br>2		FLLC
55.	Do you fish with short/no lower body clothing? ***(Wo ntaadeɛ a wohyɛ wɔn fɔm de yi nam no yɛ a na yɛ tia anaa wonhyɛ bi koraa?)***	Yes (Aane)<br>No(Daabi)	1<br>2		FSLC
**BATH (HYGIENE)**					
56.	Do you have your bath for hygiene? ***(Ahoteɛ nti na yɛ a wodware?)***	Yes (Aane)<br>No(Daabi)	1<br>2		BATH
57.	Do you have your bath for hygiene, but not in the Densu river? ***(Ahoteɛ nti na wodware nanso ɛnyɛ Densu nsuo mu anaa?)***	Yes (Aane)<br>No(Daabi)	1<br>2		BAH

58.	Do you have your bath for hygiene in the Densu river? ***(Ahoteɛ nti na wodware wɔ Densu nsuo no mu anaa?)***	Yes (Aane)<br>No(Daabi)	1<br>2		BATD
59.	Do you have your bath for hygiene, but not water from open borehole? ***(Ahoteɛ enti na wodware nanso ɛnyɛ wɔ nsubura mu nsuo mu?)***	Yes (Aane)<br>No(Daabi)	1<br>2		BNBH
60.	Do you have your bath for hygiene with water from open borehole***?(Ahoteɛ enti na wodware nanso ɛwɔ nsubura mu nsuo mu?)***	Yes (Aane)<br>No(Daabi)	1<br>2		BOBH
61.	Do you Swim/dive/play in water? ***(Wo di agorɔ, dware, to wo ho to nsuo mu?)***	Yes (Aane)<br>No(Daabi)	1<br>2		SIW
62.	Do you Swim? (***Wonim nsuo mu adware?)***	Yes (Aane)<br>No(Daabi)	1<br>2		SWIM
63.	Do you Swim, but not in the Densu river ? ***(Wonim nsuo mu adwareɛ nanso ɛnyɛ Densu nsuom?)***	Yes (Aane)<br>No(Daabi)	1<br>2		SWND
64.	Do you Swim in the Densu river? ***(Wodware wɔ Densu nsuo no mu?)***	Yes (Aane)<br>No(Daabi)	1<br>2	>> 64a	SWID
64a.	If yes, How often do you swim in the Densu river in a week	__________			
